# Religion, Health, and Life Satisfaction Among Somali and Gambian Women in Norway

**DOI:** 10.1007/s10943-022-01561-1

**Published:** 2022-04-27

**Authors:** Inger-Lise Lien

**Affiliations:** Section for Trauma Catastrophes and Forced Migration – Adults and the Elderly, Norwegian Centre for Violence and Traumatic Stress Studies, Gullhaugveien 1-3, 0484 Oslo, Norway

**Keywords:** Gambian and Somali women, Female genital mutilation/cutting, Trauma, Religious feeling guidelines, Satisfaction of life, Norway

## Abstract

This article describes and analyses the religious justifications for the life satisfaction reported by two groups of Muslim women. Approximately, twenty Somali women and twenty Gambian women, living in Norway, who had experienced trauma and pain due to female genital mutilation/cutting as well as other traumas and hardships, were interviewed. While the Somali women adhere to conservative Islam and try to cope with their life through endurance and patience, the Gambians belong to a Sufi tradition and verbalise their dissatisfaction in order to receive help from Sufi saints. Therefore, there are two religious codes, here called emotionologies, within the Muslim tradition that have different impacts on the expression of life satisfaction and women’s ways of coping with pain and suffering.

## Introduction

This article describes how Gambian and Somali women in Norway cope with their life and health problems with support from their religion. The women in this study, all of whom are Muslims, experienced the procedure of female genital mutilation/cutting (FGM/C) during childhood. In their adult life, some women live with mental and physical consequences of FGM/C that can affect their sexual life and relationships, result in chronic pain, and sometimes leading to divorce or to women becoming part of polygamous marriages. In spite of many hardships, some of the women studied scored very high on the life satisfaction scale. Religion can provide guidelines for coping with suffering and trauma, leading to better life satisfaction in spite of torment. This article analyses the different attitudes towards life and suffering held by Gambian and Somali women with the help of religious guidelines and concepts belonging to a conservative Muslim tradition and the tradition of Sufi Islam.

According to Silvermark et al. (2008), life satisfaction is the most common measure of wellbeing found in the literature. The focus in many studies of wellbeing has been on income and marriage relations, but according to Kahneman et al. (2006), it is an illusion that higher income alone leads to higher life satisfaction. According to Krause & Wulff, (2005), social networks and good health are the major sources of wellbeing. Some studies have found a connection between suffering pain and low life satisfaction (McNamee & Mendolia, 2014). Chronic pain is associated with poor health conditions, disability, decreased participation in the labour market, and lower quality of life. Chronic pain seems to be more common in women than in men (Bergman et al., 2001). One study found that women with FGM/C are less satisfied with life and marriage than women without FGM/C are (Khodabakhshi Koolaee et al., 2012). Other studies have shown that married women are more satisfied than unmarried women but women in polygamous marriages are less satisfied with life than women in monogamous marriages (Al-Krenawi, 2012).

Religion seems to be central among the factors that play a role in life satisfaction (Lim & Putnam, 2010). The explanation for this appears to be an attachment to social networks within the congregation as well as sharing a strong religious identity with other members. Trust in a divine power and the feeling of love from God add to life satisfaction (Greeley & Hout, 2006). Religion appears to be less important than health and loneliness but more crucial than education, race, and gender in providing life satisfaction (Lim & Putnam, 2010).

According to Green and Elliott (2010), there is no evidence indicating that believing in Jesus versus worshipping Allah have differential health effects, but these researchers suggest that ideological underpinnings of religious beliefs may affect health. Sharma and Singh (2019) conducted a study among respondents from six major religions in India and found that the virtues of forgiveness, gratitude, and altruism promoted wellbeing among their religious respondents. Other researchers (Akhtar et al., 2017) have also found a positive link between forgiveness and wellbeing. This recommends a closer examination of what ideas and guidelines that exist within the scripture of a religion that promote positive virtues. Can religion provide specific regulatory tools for expressing feelings, resembling what Hoschield (1979) describes as feeling management? Hoschield holds that there is a distinction between emotion and emotion management and that feeling rules specify the intensity, direction, and duration of emotions. According to Hoschield, rules and guidelines are sometimes the opposite of real feelings but can have an impact on real feelings and the individual’s inner self (Hoschield, 1979).

Because of this distinction between emotions as they are felt and emotions as they are expressed, Stearns and Stearns (1985, 1986) developed the concept of emotionology, which is ‘the attitudes or standards that a society, or a definable group within a society maintains towards basic emotions and their appropriate expression’ (Stearns & Stearns, 1985, p 813).

Thereby, religion can also contain an emotionology offering emotional guidelines and support by providing people with feeling rules and feeling management with ideas and instructions for stress reduction through contemplation, meditation, and prayers. According to Krause and Ellison (2003), forgiveness in particular is positively correlated with greater life satisfaction, and forgiveness is mentioned in several verses in the Bible such as Matthew 6:12. These instructions are specific to a Christian emotionology, but other religions also promote forgiveness. During pilgrimage to Mecca, there is a special forgiveness day. Forgiveness and repentance are very important instructions for Muslims and are mentioned in several verses in the Qur’an (3:159, 15:85, 24:22, and 42:40). There is a growing awareness among researchers of the role of religiosity in healing, describing how Muslim individuals use their religious leaders and shrines to solve emotional problems (Mitha, 2019; Charan et al., 2018). Isgandarove (2019) has shown that *Muraqaba*, a Sufi meditation technique, has some similarities to psychotherapy. The religion of Islam may thereby have an inbuilt religious emotionology that contains ideas and guidelines for feeling management, healing, and dealing with hardships and pain that can have a direct or indirect impact on believers’ life satisfaction and wellbeing.

## Coping with Female Genital Mutilation/Cutting

The Somali and Gambian women interviewed in this study have experienced FGM/C, which is a tradition widespread throughout Sub-Saharan Africa as well as some Arab and South East Asian countries (WHO, 2008). Through migration, the practice has spread to many Western countries as well (Johansen, 2002; Johnsdotter, 2020; Schultz & Lien, 2014; Ziyada et al., 2020). Do the negative health consequences of the procedure lead to low life satisfaction for the women who have been cut?

A systematic review of gynaecological consequences of FGM/C (136 studies) found severe health complications including clitoral inclusion cysts and urinary problems such as urinary tract infections, dribbling, and poor urinary flow as well as painful menstrual periods and cramps and accumulation of blood in the uterus and vagina (Berg et al., 2014; Dirie and Lindmark, 1992). Another systematic review included psychological and sexual consequences and found that women with FGM/C experienced increased pain and reduced desire and pleasure during sex (Berg et al., 2010).

Somali women have experienced the most pervasive form of FGM/C (type 3 according to the World Health Organisation’s typology; WHO, 2008). Gambian women have experienced a milder version of cutting (types 1 and 2) because they have generally not had their labia stitched. For both groups of women, long-term health problems related to FGM/C become more apparent during and after puberty, when menstruating, and when married, and FGM/C may also affect child birth and sexuality and have sociopsychological consequences (Berg et al., 2010), thus representing a chronic and multifaceted health problem for women.

How do Somali and Gambian women cope with pain and suffering? Jacobsen et al. (2018: 1) conducted a study among Somali Canadian women and found that they experienced pain and discomfort due to FGM/C throughout their adult lives. However, they were intent on not giving pain any power and considered themselves healthy. When discussing FGM/C, they presented it as a normal event that is a necessary gateway to womanhood. If pain or other sensations related to FGM/C and other traumatic experiences were mentioned, the women would start laughing, behaving joyfully, avoiding the topic, normalising it or saying, ‘I feel fine’.

Can this tendency to laugh and normalise both physically and psychologically painful experiences be explained by religious factors? Are there cultural and/or religious guidelines and codes that teach Somali and Gambian women to approach life challenges, pain, and suffering in ways that create positive life satisfaction? Do Somali and Gambian women have special ways of coping with suffering that constitute a positive code for survival that they find within their religion and to which they adhere, which leads to a high life satisfaction score? This article addresses these questions.

## Materials and Methods

This study is based on a mixed method design (McKim, 2017) with both qualitative and quantitative methods being used through data collection in the Oslo area. Approximately, twenty Gambian women and twenty Somalian women were interviewed using an interview guide to allow the women to first give information about characteristics, about the number of traumatic events experienced, describe their life situation, both past and present, as well as their experiences with FGM/C. A list of potential traumatic events was created based on the event list from the Harvard Trauma Questionnaire (Jakobsen et al., 2007; Mollica et al., 1992), and women were asked to list how many events they had experienced. The potential traumatic events to which they could have been exposed were lack of food, military combat, rape and sexual abuse, murder of family and friends, forced isolation, kidnapping, almost being killed, serious physical injury, violence, head injuries, and FGM/C. FGM/C was added to the list because some studies have found that it leads to traumatic symptoms (Köbach et al., 2018; Knipscheer et al., 2015).

According to the Diagnostic and Statistical Manual of Mental Disorders-5 (American Psychiatric Association [APA], 2013), a traumatic event is ‘exposure to actual or threatened death, serious injury, or sexual violence either directly or indirectly witnessing the event or learning about it.’ In sum, there were 24 potentially traumatic events that the interviewed women could have experienced. There was no intention to diagnose symptoms of posttraumatic stress disorder (PTSD). Rather, the purpose was to add the number of potential traumatic events to which the women in the two groups could have been exposed. The women were also asked, ‘On a scale from 1 to 10 where 1 is the worst level of life satisfaction and 10 is the best, where would you say you are today?’ The women were then asked about their hopes for the future 2 years from now.

One Gambian woman, an activist, was a gate opener in the Mandinka Gambian community and conducted half of the interviews herself. The women interviewed are part of a network who are friends and usually meet together once per month in a community house, bringing their children along. The researcher visited these women in their homes for the rest of the interviews and participated in Eid celebrations and other gatherings that the women organised. Additionally, a Somali midwife in her 50 s assisted in recruiting and interviewing Somali participants. She conducted approximately half of the interviews, and the researcher conducted the other 10 interviews. The group of Somali interviewees was not as closely knit as the Gambian interviewees. They belonged to several clans, and some of them did not know each other even though they all knew the assistant researcher. Confidence and trust had been established over the years with some of the Somalis and most of the Gambians through earlier research projects in which some of the same women had participated. This way of recruiting informants is based on a snowball sampling method or a response-driven sample common in social anthropology and sociology that is often used to study difficult-to-reach populations (Gile & Handcock, 2010). In this study, the network lines in the Gambian and Somalian networks that surrounded the research assistants were followed.

The interviews started with the quantitative part summarising the traumatic events and the level of life satisfaction for each of the two groups of women. Thereafter, the study proceeded with a qualitative design using open interviews, allowing topics to come up spontaneously. A participant observation model was also included as the researcher became integrated in the participants’ personal networks and participated in the different community and religious functions that were held, obtaining insight and information through participation. Below is a table (Table 1) showing the methods used during interviewing Somali and Gambian women as well as marking the time with a cross (spring and autumn) when the different methods were used.

**Table 1:** Qualitative and quantitative methods with timeline

Methods	2017		2018		2019		2020	2021
*Open interview guide including*								
Quantitative								
Characteristics								
HTQ-events								
Satisfaction of life scale								
Qualitative								
Open guideline talk								
Somalis				x	x	x		
Gambians			x	x	x			
*Participant observation*								
Qualitative								
Somalis				x		x		
Gambians		x	x	x	x	x	x	
*Telephonic interview about religion*								
Qualitative								
Islamic experts								x
Somalis							x	
Gambians							x	

A telephone round with 10 key interviewees in both groups was conducted in 2020 and 2021 to discuss the role of religion in coping with the hardships of life and FGM/C. The reason for this was that religious concepts and explanations arose during the open interviews and during participation. Religious experts connected to two mosques were called to discuss concepts in Islam. The telephonic communication was due to the COVID-19 pandemic, which made meeting face-to-face difficult. A marabout in the Gambia was interviewed several times using WhatsApp.

All participating women received both oral and written information about the project and signed a letter of consent after being informed about anonymity and the study’s ethics clearance. The interviews lasted up to two hours each; several women were interviewed twice and some three times. Interviews were not audio recorded since the women were negative and sceptical about recording. Handwritten diary notes were taken during the interviews and participation and later typed. The Regional Committee for Medical and Health Research Ethics in Norway approved this study on 12 September 2017.

## Results

### Life Satisfaction and Life Problems Among Somali and Gambian Women in Norway

#### Somali Women

As Table 2 shows, the Somali women in this study had an average age of 43; the oldest woman was 68. They had lived in Norway for more than 10 years on average, which is less than the Gambians’ average of 17 years (Table 2). The Somali women belonged to different networks. Some belonged to the same family group, others met once per week for physical training, and still others seemed not to be part of a particular network among the interviewees. Most of the Somali women did not have a job and lived on social welfare. Of the six Somali women who had jobs, four worked in the health sector as nurses, a midwife, and a cleaner, while two worked in childcare. Of the Somali women, six were married, while most of the Gambian women (as many as 16) were married. Some of the Somali women stated that they had been married two or three times but were now divorced and lived like single mothers with children. When going to the mosque to pray, most of them used the Tawfiiq Islamic Centre in Oslo, which is a conservative mosque.

**Table 2:** Overview of participants’ characteristics

Characteristics	Gambian women	Somali women
Number of participants	20	20
*Age*		
22–27	1	5
28–33	2	2
34–39	6	1
40–45	3	2
46–52	5	3
52–57	2	2
58–62	0	4
63–70	1	1
Average age	41	43
*Social status*		
Married	16	6
Unmarried	1	4
Divorced	3	8
Widowed	0	2
Average length of stay in Norway	17.2 years	11.2 years
< 1 year	0	1
1–5 years	2	2
6–9 years	1	3
10–14 years	4	3
15–19 years	5	8
> 20 years	5	1
*Employment*		
Yes	12	6
No	6	12
Student	2	2
*Type of FGM/C*		
Type 1	8	3
Type 2	7	
Type 3	4	13
Type 4		1
Missing information	1	3
Average number of traumatic events experienced	1.7	8.4
Average life satisfaction score now	4.1	6.8
Average life satisfaction score 2 years from now	6.0	9.3

On average, the Somali women had experienced 8.4 traumatic events from the list of traumatic events, which also included FGM/C. In spite of this high number of traumatic events, the women seemed joyful and happy and held that their life satisfaction is better than average. On a scale from 1 to 10, Somali women’s life satisfaction was generally above 6. Only one Somali woman out of 20 had a life satisfaction score below 5. Out of 20 women, six rated their life satisfaction between 8 and 10. When it came to hopes for the future, 13 of the women hoped that 2 years from now, their life satisfaction score would be 10. The Somali women therefore seemed to have a very optimistic view of their future. When asked about FGM/C and health, one woman explained that ‘Somali women will not complain about their genitalia, but if they have pain, they will talk about it as back pain’. As another woman said, ‘Genital pain is the law of the woman’.

The Somali women’s assessment of life now and in the future indicates that they positioned themselves remarkably high on a scale measuring satisfaction and hopes for the future. This was also the case for those who had experienced several traumatic events and suffered pain due to FGM/C. This tendency to assess their life satisfaction so positively is in line with findings in a report by Statistics Norway (Barstad, 2018). The average Somali score on the quality of life instrument used in the study was the highest of all the studied groups. The majority population, namely ethnic Norwegians, scored 8.1 on the scale, while the Somali score was on average 8.8 on a scale from 0 to 10 even though they scored low on indicators of employment and high on indicators of poverty (Vrålstad & Wiggen, 2016).

The tendency towards positive thinking among the Somalis is illustrated by the following case: A Somali woman had been diagnosed with PTSD and given treatment for trauma symptoms by psychiatrists in Norway after having been raped by several men in a Middle Eastern prison. She explained, ‘One tried to suffocate me when I cried. They raped me every day’ (Somali, age 22). She was still receiving treatment during the interview period. However, when asked about her life satisfaction on a scale from 1 to 10, she rated her life satisfaction at 7, and she expected it to be at 10 in 2 years. She expressed satisfaction and hopes for the future in spite of struggling with mental health problems. This positive thinking is also shared by other Somali women. There may be a relief in having escaped from a traumatic situation in the past behind the young woman’s life satisfaction, but she also provided a religious justification for this high degree of life satisfaction, saying, ‘As long as you believe in God, you will get a better life.’

It seems that the Somalis try to maintain a happy expression and endure pain and suffering. They were not willing to discuss their physical or psychological pain or sexual problems at length. As one of them said, ‘To be a woman, you must expect pain and endure it’ (Somali, age 44). Another woman said, ‘You must never say that you have pain in your genitalia area. You have to endure it. To have pain is to be a woman’, and another, ‘We are very proud people, so we do not complain.’

#### Gambian Women

The Gambian women were on average 41 years of age, and 16 out of 20 were married (see Table 2). They were Mandinkas who had a sense of togetherness because they used to meet once per month in a community house in Oslo. This implies that their network was functional and effective and provided them with an identity as Mandinka women living in Oslo. Most of the Gambian women worked (see Table 2) and lived in apartments they owned with their husbands. They called each other ‘darling’, ‘sweetheart’, and ‘beautiful’ and seemed happy and joyful in everyday life. They regarded themselves as integrated women who are modern, open, and tolerant; they did not use hijabs in everyday life but rather wore wigs. They also used to go to mosques for different functions and bring food to eat as well as arrange picnics in the park during the summer, in which the researcher used to participate. However, their life satisfaction scores were much lower than those of the Somali women. When asked about their life satisfaction on a scale from 1 to 10, most of the Gambian women placed themselves below the middle with an average of 4.1 compared to the Somalis’ 6.8 (see Table 2) even though from an outside perspective they seemed well-situated and happy. When it came to hopes for the future, on average, the Gambians expected their life satisfaction to be 6 in 2 years, while the Somalis’ projected score was on average above 9 (see Table 2). The Gambian women’s hopes for the future as measured in this study were therefore much lower than the Somali women’s scores.

The Gambian women had all experienced FGM/C, which they described as a very frightening event that happened during childhood. They were not reticent to discuss their pain and suffering. Many of them said that they suffer sexual consequences of FGM/C because they have a great deal of pain in the vaginal area and sex is painful. Some were afraid that their husband would find a second wife. A few women were dissatisfied because they had discovered that their husbands had already taken a second wife in the Gambia. None of them had been in contact with a psychologist or received a diagnosis of PTSD. On average, they had experienced 1.7 traumatic events compared to the Somalis’ 8.4 (see Table 2). When asked about reasons for their low life satisfaction score, one woman said, ‘Many women get depressed when they get information that their husband has one or more additional wives. They feel betrayed.’ During the interviews, it became clear that three women were in the process of separating from their husbands for this reason. Two women left their husbands during the data collection period, and one left but later changed her mind because she said that she loved her husband too much and took him back even though he had married another woman in the Gambia. ‘Men try to establish their second family in the Gambia. The women have to accept this because it is a religious right that the man has, but the women are very unhappy sharing their husband. It destroys their marriages.… This polygamy is the reason why they are so depressed and do not have hopes for the future’ (Gambian, age 50).

The women linked polygamy to religion because they see it as a right that men have in Islam, which they are not ideologically allowed to disapprove of even though it makes them unhappy. During interviews, they expressed disapproval of the tradition, and a group of Gambian women arranged a seminar during which polygamy was discussed and criticised. They also seemed to link FGM/C to pain and a lack of sexual desire as well as to marriage problems, all of which made the women dissatisfied with life. Some of the Somali women also seemed to link FGM/C to polygamy, and some expressed support for the tradition. As one of them said, ‘Sometimes women will ask the husband to remarry after having had children. She does not have time for a man, and she thinks that it is okay that he takes another woman, especially if she has genital pain’. Another woman said, ‘Some women feel that polygamy is a relief; then they do not need to have sex’ (Somali, age 35).

Chronic health consequences due to FGM/C thereby seem to affect marriage relations and lead to men taking second wives, thus indirectly contributing to low life satisfaction. However, dissatisfaction may not necessarily be expressed, which may be an effect of how religion plays into the women’s interpretations of their life situation.

## Explanations of Life Satisfaction Based in Religion

There are at least two types of emotionologies connected to Islam that can impact ways of enduring hardships, pain, and problems as well as women’s life satisfaction. One is the Sharia type of Islam represented by a more fundamentalist interpretation of the Qur’an and the Hadith. The other is Sufism, which allows for a divine representative on earth operating between men and God. Sufism is widespread in the Gambia, and although Somalia also used to have a dominant Sufi tradition (Lewis, 1998), Somalia has apparently been pervasively influenced by conservative versions of Islam like Wahhabism and Salafism in recent years. Sufi mysticism has been criticised from many perspectives, and the Somali interviewees generally expressed a negative attitude towards Sufism, saying, ‘It is superstition’ and ‘We do not believe in it’. When troubled, Somali women often go to the mosque to pray or invite the imam to their home for prayers. The imam is considered an ordinary person who leads prayers in the mosque and has great knowledge about the Qur’an; he is not viewed as a divine power who can change people’s destinies like the Sufi saints are. When in trouble, the Gambian women call their Sufi saints, the Marabous, in the Gambia and go to the mosque to pray. For both groups of women, religion is a place to seek comfort and advice when feeling pain and unhappiness.

Key Somali interviewees explained the reasons for the women’s high scores on the life satisfaction scale by mentioning two religious concepts: *ajr* (reward) and *sabr* (patience/endurance). One woman explained, ‘You lose *ajr* when you say that you are not satisfied. If you believe in God, you must believe that your destiny is God’s will’ (Somali, age 55). An older Somali woman explained, ‘Your life is a test that you must endure. If you complain, you complain about God. It means you do not believe in God’ (Somali, age 63). Other women added, ‘If you don’t believe in your future, you don’t believe in God’ (Somali, age 35) and ‘If you endure the tests that God puts you through, then you will get forgiveness from God, and mercy, and go to Paradise’ (Somali, age 65).

*Sabr* and the fear of not receiving *ajr* are both part of what this article terms a religious emotionology for Somali Muslims and contain emotional guidelines. According to the interviewees, these concepts have a basis in the Qur’an (Surah 11, 39:10, 35:75), are used in everyday conversation and are said to be very important and even commandments. To have *sabr* is said to ease suffering and reduce anxiety during and after traumatic events and hardships. In addition, it will benefit the person on Judgment Day because *sabr* will provide the blessing and forgiveness from God necessary for the reward (*ajr*) needed to gain Paradise. Complaining about pain and suffering seems to indicate a lack of *sabr* as well as a lack of faith, which can lead to a lack of *ajr.* Individuals are advised and instructed to express and develop feelings of satisfaction, avoid dissatisfaction, and have faith in God. ‘We are taught not to complain. We are taught to have positive thinking and believe in God’ (Somali, age 55). ‘Even though you are poor and unhappy, you must thank God for your destiny and say “Alhamdulillah” [all praise and gratitude belong to Allah]’(Somali woman, age 45).

This emotionology advises Somali women to have faith and trust in God, have patience, and not express dissatisfaction or be dissatisfied even though they suffer pain due to FGM/C or other problems in life. This religious emotionology can be a factor that explains the high life satisfaction score among the Somalis found in both this study and the SSB study (Barstad, 2018).

The Gambians living in Norway adhere to Sufi Islam, which teaches an alternative way to God’s blessing (Baldick, 1989; Ernst, 1985; Esposito, 1999; Trimingham, 1971). The living Sufi saints represent an intermediate spiritual power between God and people and have developed different methods of meditation and rituals for achieving oneness with God called F*ana ul Allah*. Isgandarova (2019) has argued that the Sufi techniques for meditation reduces stress and could be integrated into psychotherapeutic treatment. The Sufi saints, who in the Gambia are called marabouts, are thought to have special spiritual powers and able to influence God through their prayers and improve people’s destiny and suffering. When troubled, the Gambians in Oslo contact their marabouts in the Gambia for help, and ask for rituals like sacrificing a goat or prayers in order to change their destiny. Gambians have knowledge about the necessity of *sabr* since this is a very important Islamic concept. Like the Somalis, they believe that God decides their destiny before birth, but they also have a belief that their destiny can be changed with the help of a marabout. ‘You have to have good morality, never maltreat people, and you must give from your heart, and if you repent, God will forgive you and ease your suffering. We think we can go to the marabout for juju [amulets] and prayers so that our problems can be lifted’ (Gambian, age 47). Another woman said, ‘Persons can and should inform the marabout about their suffering and thereby get help to solve their problems’ (Gambian, age 40). Forgiveness is thought to improve life and produce rewards. The prayers of the marabout are also thought to have a stronger weight to improve the individual’s life than the prayers of the ordinary believer do. The positive effect of contacting the marabout is evident in the example that follows.

When Awa discovered that her husband married a second Gambian woman in the Gambia, she was very unhappy and filed for a divorce. The divorce process was difficult, and she quarrelled with her husband about financial issues. She obtained sick leave for 6 months because she was very depressed. She contacted a marabout in the Gambia, who called her every day on WhatsApp and calmed her down, saying that he had prayed for her all night to improve her destiny, provide blessing and remove evil forces around her.

A marabout functions as a kind of community psychologist and social worker. In addition to therapeutic prayers, he follows up on the progress of his disciples who live in foreign countries like Norway via telephone and WhatsApp. The fact that the marabout checked on Awa almost every day via phone calls gave her support, strength, and hope. In order to receive this kind of help from the marabout, Awa had to formulate her unhappiness, dissatisfaction, and problems for him in order to receive support. These alternative methods of influencing God make it possible for Gambian women to be more open in expressing suffering and dissatisfaction without being suspected of disbelieving in God. Feelings of dissatisfaction allow for opportunities to receive help from a marabout, who addresses God through prayers on the woman’s behalf. Acknowledging pain and suffering thus becomes a first step towards searching for help within the religious sphere.

## Discussion and Conclusion

There are at least two types of religious structures within Sunni Muslim communities, one of which is the more conservative Sharia-based Islam, while the other is the Sufi tradition. Within Muslim countries, these two traditions often exist side by side, but in recent years, there has been an intellectual call to return to the basic scripture and reject Sufism, which some view as based on superstition rather than on religious facts within books. This criticism of Sufism is strong among conservative Muslims and those who have embraced the Wahhabi and Salafi types of Islam, which are more prevalent today among Somalis in Norway, who used to have a strong Sufi tradition. More fundamentalist Islam has not yet reached the Gambians to a high degree, and the institution surrounding the Sufi saints remains strong in the Gambia as well as among several other Muslim groups living in Norway.

In addition to the institutional focal points within the religion like the imams and the marabouts, there are the concepts and guidelines within the scripture that provide advice about the management of feelings and life problems. According to Thomas et al. (2018), *sabr* is mentioned more than 70 times in the Qur’an and connected to the concept of acceptance ‘in that one sees the situation as a test/trial from God through patience’ (Mitha, 2019: 197). Both literate and illiterate Muslims know the meaning of the concept and what it implies. ‘We will surely test you with something of fear and hunger and a loss of wealth and lives and fruits, but give good tidings to the patient, who when disaster strikes them, say, “indeed we belong to God and indeed to him we will return”’ (Surah 2:155, 156, The Qur’an [edited by al-Mehri 2021] page 46). Enduring hardships and pain will lead to *hasanat* (credit) when good and evil deeds are counted and weighed against each other on Judgment Day.

The Somali women in this study had experienced several traumatic events in their past, averaging 8.4 traumatic events compared to the Gambian average of 1.7 traumatic events. The Somali women had also experienced the most pervasive form of FGM/C (type 3) that can potentially cause more chronic health problems than types 1 and 2 do (WHO, 2008). However, in spite of many gruesome experiences with which they struggle as well as pain due to the FGM/C procedure, the Somali women expressed a high level of life satisfaction. This attitude of endurance and patience prescribed in the religion can lead to resilience against trauma symptoms and depression but also result in resistance and barriers to contacting health services when Somali women have symptoms of mental health problems or pain from FGM/C. The religious instructions or the emotionology and the cultural traditions connected to it are important for health personnel to understand in order to provide good health services to those who have experienced traumatic events or ongoing pain, depression, and life problems. It can be necessary for health personnel to take more time with their patients and look beyond declarative statements of life satisfaction to see if these declarations signify actual feelings or are merely a way of communicating a strong belief in God.

Marriage problems as well as health problems and pains are reasons for Gambian women to contact their marabout for amulets, herbs, and prayers. Marriage problems and the chronic pain these women suffer are the main reasons they give for their dissatisfaction with life. The Sufi saint or the marabout within Sufism is an intermediate religious force between humans and God who has the role of an advocate on Judgment Day and is believed able to influence people’s destiny. This force has softened the interpretation of some religious concepts and opened a way for people to improve their destiny. This implies that people must formulate their dissatisfaction before the intermediate power. These opportunities make their religious philosophy of feeling, or their emotionology, less imperative, less frightening, and softer and allow for feelings of dissatisfaction to be expressed.

These two different traditions within Muslim communities are important to be aware of when providing mental and physical help to women and men who have a strong belief in the Muslim religion and in the necessity of *sabr*, which can give people a stoic attitude towards life. The religious emotionology can also remove or weaken the feeling of guilt often present in individuals who suffer by externalising the cause for suffering, seeing it as a test from God. This form of meaning-making within the religion can make the psychological burden of a trauma easier to carry because it puts responsibility on the divine power.

Both groups of women thereby have their own separate coping mechanisms, and their strategies of coping work in opposite directions. (1) Somalis are inclined to suppress dissatisfaction, try to be satisfied and express satisfaction, think positively, and exercise endurance in order to trust God and be rewarded on Judgment Day. (2) Gambians are allowed to express dissatisfaction when enduring hardships in order to formulate their problems for a Sufi saint, asking him to call upon God through prayers. The two types of coping mechanisms can also have some drawbacks when professional help from mental health services is to a high degree avoided in favour of Sufi saints in the Gambia or too much endurance by Somalis in order to follow the scripture. Hence, it can lead to people suffering the consequences of trauma and waiting too long before expressing their problems to health services representatives.

On the other hand, if it is difficult to obtain access to mental health services in the West, as some studies (Ziyada and Johansen, 2021; Lien & Hertzberg, 2020) have described, it is helpful that the Somali and Gambian women have their own religious traditions to turn to. These traditions may offer kinds of treatment and help to persons suffering traumas and depression. It is important that further studies investigate the effects of these traditional treatments. Only through systematic research will it be possible to understand the interference between treatments based on science and treatments based on religion and culture.

It is important for health workers to be familiar with the emotionologies surrounding patients in order to understand what is at stake for their patients and which moral and emotional codes their patients live by. Therefore, students of medicine and mental health should be taught forms of cultural and religious emotionologies as well as institutional alternatives within religion. This knowledge would give health workers not only an emphatic understanding but also a basis from which to build and offer treatments that can take their patients’ motivational sources into account. Only by understanding patients’ religious and cultural contexts can healthcare professionals develop and offer effective and relevant treatment that builds upon science but also takes patients’ meaning-making codes into account. Scriptures can also provide ideas to build upon when helping women who suffer from traumas and depression caused by the procedure of FGM/C, marriage problems, polygamy, and other types of hardships in everyday life.

### Study Limitations and Strengths

In terms of the representativeness of each sample, this study would have benefitted from larger samples of women from Somalia and the Gambia living in Norway. On the other hand, a small, response-driven sample based in a dense network of women can provide insights into background factors that create patterns of differences between the two groups of women, such as differences in religious understandings of dealing with suffering. The impact of religious ideas and practices would not have been possible to discover without an open research design accessing informal situations in which women freely discuss their religious attitudes, marriage problems, and pains due to FGM/C. This is the strength of the study. However, the COVID-19 pandemic created limitations to the last stage of information gathering, especially to the opportunity to get more in-depth information through participant observation about the way Somali women use the imams and the mosques to deal with pain and suffering.

## Data Availability

The corresponding author confirms that the data supporting this study are available within the manuscript. The corresponding author can be contacted for further information about availability.
